# Identifying Alzheimer's disease genes in apolipoprotein E^−/−^ mice brains with confirmed *Porphyromonas gingivalis* entry

**DOI:** 10.1177/25424823251332874

**Published:** 2025-03-31

**Authors:** Sim K Singhrao, Claudia Consoli

**Affiliations:** 1 School of Medicine and Dentistry, University of Central Lancashire, Preston, UK; 2 Central Biotechnology Services, College of Biomedical and Life Sciences, Cardiff University, Wales, UK

**Keywords:** Alzheimer’s disease, apolipoprotein E, genes, inflammation, *Porphrymonas gingivalis*

## Abstract

**Background:**

The apolipoprotein E allele ε4 is the most well-known predisposing genetic risk factor for Alzheimer's disease (AD).

**Objective:**

To identify AD genes in apolipoprotein E^−/−^ (ApoE^−/−^) mice brains with confirmed entry of *Porphyromonas gingivalis*.

**Methods:**

TaqMan™ Mouse AD arrays were performed on orally infected ApoE^−/−^ mice with confirmed *P. gingivalis* entry and compared with sham infected mice brains (N = 4) at 12- and 24-weeks post infection.

**Results:**

Gene expression by qPCR demonstrated that in the *P. gingivalis* 12-weeks post oral infection, two genes were statistically significantly changed in their expression. These were cyclin dependent kinase 5 regulatory subunit 1 (Cdk5r1, 0.15 logfold change, p = 0.05) and Interleukin 1 alpha, (IL1a, −0.10 log fold change, p = 0.012). In the *P. gingivalis* 24-weeks post oral infection, three genes were statistically significantly changed in their expression. These were cholinergic receptor nicotinic alpha 7 subunit or Chrna7 (0.10 log fold change, p = 0.02), mitogen-activated protein kinase 1 or Mapk1 (0.10 log fold change, p = 0.05) and visinin like 1 or Vnsl1 (0.01 log fold change, p = 0.04). 87 out of 92 AD target genes demonstrated no difference between infected and sham mice brains.

**Conclusions:**

Five genes, from a recognized AD panel had statistically significantly altered expression in the ApoE^−/−^ mouse AD model following *P. gingivalis* entry into the brain. This suggests the ApoE^−/−^ genetic variation may control the biological activity of specific genes relevant to inflammation and neuronal plasticity following *P. gingivalis* infection.

## Introduction

Periodontal disease(s) are caused by the dysbiosis of a polymicrobial oral microbiome, which activates the host's local innate, systemic and organ specific immune responses^1^ destroying the hard and soft tissues in the oral cavity, the severity worsening with advancing age. One of the most important microbes associated with periodontal disease is *Porphyromonas gingivalis*, a keystone bacterium associated with severe generalized periodontitis.^2^ Accordingly this pathogen is the primary choice for inducing experimental periodontal disease in rodent models of human disease, including the Apolipoprotein E (ApoE^−/−^) mouse.^3,4^ The ε4 allele of the susceptibility gene *APOE* encoding apolipoprotein E (ApoE) is the strongest genetic risk factor to date for both familial and the sporadic forms of AD.^5,6^ The functional ApoE protein, plays a crucial role in lipid metabolism and the immune system. Its loss in function greatly hinders the body's ability to mount an effective inflammatory response to infections, and explains why the ApoE^−/−^ rodent model of human AD is prone to bacterial infections.^4^ Consequently, the ApoE^−/−^ mouse model has become a critical research tool in investigating the relationship between periodontal infection and its contribution to the pathophysiology of systemic diseases^7–11^; AD,^8,11–14^ cardiovascular disease and type II diabetes.^15–17^

AD is characterized by progressive cognitive impairment associated with neuronal loss, microglial activation and typically the accumulation of extracellular amyloid-β (Aβ) plaques and neurofibrillary tangles (NFTs) composed of paired helical filaments of hyperphosphorylated cytoskeletal protein, tau. Inheriting the *APOE* ε4 allele increases the severity of AD pathology because this gene predisposes individuals to infections by compromising the hosts immune system,^18–20^ and disturbances in the lipid metabolism.^21^

There is a well described association between systemic infection such as urinary tract and pneumonia (and periodontitis) with exacerbations of cognitive decline in AD associated with changes in the host's inflammatory response to the presence of systemic pathogens.^22^ Furthermore, the presence of *APOE* ε4 has been implicated in an aggressive form of periodontitis in a Chinese population.^23^ In accord with finding *P. gingivalis* in the brain tissue of patients who died of confirmed AD.^24,25^

The present study investigated the regulation of AD associated genes by using an ApoE mouse model infected with oral *P. gingivalis*. The potential relationship between infection by oral *P. gingivalis* and regulation of AD risk associated genes in a genetically modified mouse model (ApoE^−/−^) could uncover new insights into the mechanisms through which infection with this oral bacterium may contribute to AD pathophysiology.

## Methods

### Tissue resource

Eight-week-old male apolipoprotein E (ApoE^−/−^) mice strain B6.129P2-Apoetm1Unc/J (Jackson Laboratories, Bar Harbor, ME, USA) brain tissues were remnants from the orally infected and the sham treated ApoE^−/−^ mice from the Poole et al.,^12^ study at 12- and 24-week time points that were stored at −80°C in Trizol. Full description of the infection regime is fully described elsewhere.^11,12^ Institutional ethical approval for this study was obtained from the Animal Projects Committee of the University of Central Lancashire for research on animal tissues as secondary users (Ref. No. RE/11/01/SS). The only difference in the cerebral brain tissue chosen for gene analysis, in the present study was that it was selected from previously confirmed detection of *P. gingivalis* DNA by PCR (see Figure 1 in Poole et al.^12^) summarized in Table 1.

**Figure 1.**
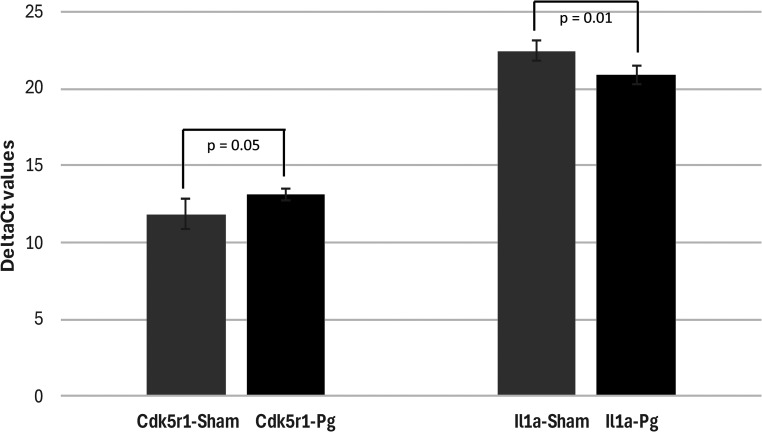
q-PCR analysis 12-weeks post infection. Of the 92 target genes evaluated in the *P. gingivalis* 12-weeks post infection the key genes that were statistically significantly changed in their expression were Cdk5r1 (0.15 logfold change, p = 0.05) and IL1a (−0.10 log fold change, p = 0.012). The expression of the two genes by q-PCR analysis (N = 4) across the sham infected (control) and *P. gingivalis* (Pg) infected was analyzed by the two variant T-Test.

**Table 1.** *P. gingivalis* DNA confirmed mouse brain specimens used for the TaqMan™ Mouse Alzheimer disease gene expression arrays.

Model and infection type	Time point	Brain number	Reference and figure number
ApoE^−/−^**Sham**-infection	12 weeks	*Brains 1–4*	Poole et al.^12^ (Figure 1)
ApoE^−/−^**Sham**-infection	24 weeks	*Brains 1–4*	Poole et al.^12^ (Figure 1)
ApoE^−/−^ ***Pg*** mono-infection	12 weeks	*Brains 1, 2, 5, and 8*	Poole et al.^12^ (Figure 1)
ApoE^−/−^ ***Pg*** mono-infection	24 weeks	*Brains 1, 2, 5, and 10*	Poole et al.^12^ (Figure 1)

### Gene expression of confirmed P. gingivalis (FDC 381) mono-infected mice brains

*Total RNA extraction.* Total RNA was extracted, from the sham and the orally mono-infected *P. gingivalis* mouse brains with confirmed *P. gingivalis* strain FDC 381 entry (Table 1). Approximately 25 mg of the cortical brain tissue was used to extract the total RNA, which was purified by using the RNeasy Mini Kit (Qiagen) according to the manufacturer's instructions. The on-column Dnase digestion was performed with the Dnase-free Dnase set (Qiagen) to eliminate genomic DNA contamination. Total RNA was suspended in 30 μl Rnase-free water and the quantity and quality of the RNA was evaluated by a Nanodrop One spectrophotometer (Thermo Scientific) and an Agilent Bioanalyzer.

*Quantitative real-time polymerase chain reaction (qPCR) arrays.* Gene expression, the TaqMan™ Mouse Alzheimer disease arrays (Applied Biosystems, purchased from Thermo Fisher Scientific, UK) were performed on aforementioned tissues. The Alzheimer disease arrays contain 92 AD associated genes and four housekeeping genes. The data was normalized to one of the more stable housekeeping genes and then compared with the sham-infected mice brain tissue at 12- and 24-week time points post infection.

*Quantitative or real time polymerase chain reaction (qPCR).* One microgram of total RNA was used to generate complementary DNA (cDNA) in a 20 µl reaction by the High-Capacity cDNA Reverse Transcription Kit (Applied Biosystems). The cDNA was diluted 1:5 and used in the qPCR reactions. A separate master mix was prepared for each reservoir of the Mouse Alzheimer TaqMan array card containing 10 µl of the 1:5 diluted cDNA, 50 µl of TaqMan Gene Expression Master Mix (Applied Biosystems) and 40 µl of Dnase/Rnase free water. Four samples were analyzed per card for the 96 associated Alzheimer disease and housekeeping genes (listed in Table 2). The TaqMan® Gene Expression Assays for Alzheimer's arrays were based on the ‘amyloid hypothesis’. The selected genes (Table 2) are involved in the amyloid-β protein precursor (AβPP) processing, which generates Aβ, and include genes implicated in multiple secondary steps of Aβ aggregation, tau hyperphosphorylation, excitotoxicity, inflammation, oxidation and microglial activation. In addition, assays for genes involved in cholesterol biosynthesis are included within the panel of 96 genes associated with AD and housekeeping genes (Table 2), due to the correlation between high cholesterol levels and an increased risk of AD.^26^ qPCR was performed using a QuantStudio 12k Flex Real-time PCR System (Applied Biosystems) and data were analyzed by the Thermo Fisher qPCR analysis software. The expression levels determined by qPCR are based on relative quantification; of four reference (housekeeping) genes that were included within the panel of Alzheimer's disease associated 96 genes within the commercial arrays are Eukaryotic 18S rRNA (18S), Glyceradehyde-3-phosphate dehydrogenase (GAPDH), Hypoxanthine guanine phosphoribosyl transferase 1 (hprt1) and Importin 8 (IP08) (See Table 2).

**Table 2.** Differential expression of genes induced by *P. gingivalis* FDC 381 (Pg) entry in the ApoE−/− mice versus the sham control groups in the cerebral region of the brain.

Gene name	Gene symbol	Type of molecule	Pg/sham No change = p more than ≤0.05
Eukaryotic 18S rRNA	18S-Hs99999901_s1	Cytoplasmic organelle **-**housekeeping gene	N/A
ATP binding cassette subfamily A member 1	Abca1-Mm00442646_m1	Membrane translocase/Receptor	No change
Acetylcholinesterase	Ache-Mm00477275_m1	Enzyme	No change
A Disintegrin and metalloproteinase domain-containing protein 10	Adam10-Mm00545742_m1	Peptidase Zymogen/alpha-secretase activity	No change
A Disintegrin and metalloproteinase domain-containing protein 17	Adam17-Mm00456428_m1	Peptidase Zymogen/TACE (tumour necrosis factor-α-converting enzyme)	No change
A Disintegrin and metalloproteinase domain-containing protein 9	Adam9-Mm01218460_m1	Peptidase Zymogen/transmembrane	No change
advanced glycosylation end-product specific receptor	Ager-Mm01134790_g1	Other	No change
Amyloid Beta (A4) Precursor Protein-Binding, Family A, Member 1	Apba1-Mm00582341_m1	Other	No change
Amyloid Beta (A4) Precursor Protein-Binding, Family A, Member 2	Apba2-Mm01312425_m1	Neuronal adapter protein	No change
Amyloid Beta (A4) Precursor Protein-Binding, Family A, Member 3	Apba3-Mm00444450_m1	Neuronal adapter protein	No change
amyloid beta precursor protein binding family B member 1	Apbb1-Mm00679084_m1	Other	No change
amyloid beta precursor protein binding family B member 2	Apbb2-Mm00802117_m1	Other	No change
amyloid beta precursor protein binding family B member 3	Apbb3-Mm00524927_m1	Other	No change
Pentraxin family of proteins	Apcs-Mm00488099_g1	glycoprotein	No change
A, gamma-secretase subunit	Aph1a-Mm00778687_s1	Protease	No change
B, gamma-secretase subunit	Aph1b-Mm00781167_s1	Protease	No change
amyloid beta precursor like protein 1	Aplp1-Mm00545893_m1	glycoprotein	No change
amyloid beta precursor like protein 2	Aplp2-Mm00507819_m1	Cell cycle	No change
apolipoprotein E	Apoe-Mm00437573_m1	Lipid transporter	No change
Amyloid precursor protein	App-Mm01344172_m1	Transmembrane receptor	No change
Amyloid Precursor Protein-Binding Protein 1	Appbp1-Mm00513907_m1	Other	No change
Beta-secretase 1	Bace1-Mm00478664_m1	Enzyme	No change
Beta-secretase 2	Bace2-Mm00517133_m1	Enzyme	No change
butyrylcholinesterase	Bche-Mm00515326_m1	Enzyme	No change
Calpain 1	Capn1-Mm00482964_m1	Enzyme	No change
Calpain Small Subunit 1	Capns1-Mm00501568_m1	Enzyme	No change
Caspase 3	Casp3-Mm01195085_m1	Enzyme	No change
Caspase 6	Casp6-Mm00438053_m1	Enzyme	No change
Cell division cycle 2a	Cdc2a-Mm00772472_m1	Other	No change
Cyclin Dependent Kinase 5	Cdk5-Mm00432447_g1	Other	No change
cyclin dependent kinase 5 regulatory subunit 1	Cdk5r1-Mm00438148_s1	Enzyme	**Upregulated**
choline O-acetyltransferase	Chat-Mm01221880_m1	Enzyme	No change
cholinergic receptor muscarinic 1	Chrm1-Mm00432509_s1	Receptor	No change
Cholinergic receptor muscarinic 3	Chrm3-Mm00446300_s1	Receptor	No change
Cholinergic receptor nicotinic alpha 4 subunit	Chrna4-Mm00516561_m1	Receptor	No change
Cholinergic receptor nicotinic alpha 7 subunit	Chrna7-Mm00431636_m1	Receptor	**Upregulated**
Casein kinase 1 alpha 1	Csnk1a1-Mm00521599_m1	Enzyme	No change
Casein kinase 1 delta	Csnk1d-Mm00503623_m1	Enzyme	No change
Cathepsin B	Ctsb-Mm01310506_m1	Lysosomal enzyme	No change
Cathepsin C	Ctsc-Mm00515580_m1	Lysosomal enzyme	No change
Cathepsin D	Ctsd-Mm00515587_m1	Lysosomal enzyme	No change
Cathepsin G	Ctsg-Mm00456011_m1	Proteolytic enzyme	No change
Cytochrome P450 family 46 subfamily A member 1	Cyp46a1- m00487306_m1	Enzyme	No change
Fetal Alzheimer antigen	Falz-Mm00626131_m1	Transcription factor	No change
Galanin And GMAP Prepropeptide	Gal-Mm00439056_m1	Other	No change
growth associated protein 43	Gap43-Mm00500404_m1	Other	No change
Glyceradehyde-3-phosphate dehydrogenase	Gapdh-Mm99999915_g1	Enzyme - housekeeping gene	N/A
Gap junction beta 1	Gjb1-Mm01950058_s1	Other	No change
Glutaminase	Gls-Mm01257297_m1	Enzyme	No change
Glutamate ionotropic receptor NMDA type subunit 1	Grin1-Mm00433800_m1	Receptor	No change
Glutamate ionotropic receptor NMDA type subunit 2A	Grin2a-Mm00433802_m1	Receptor	No change
Glutamate ionotropic receptor NMDA type subunit 2B	Grin2b-Mm00433820_m1	Receptor	No change
Glutamate ionotropic receptor NMDA type subunit 2C	Grin2c-Mm00439180_m1	Receptor	No change
Glutamate ionotropic receptor NMDA type subunit 2D	Grin2d-Mm00433822_m1	Receptor	No change
Glycogen synthase kinase 3 beta	Gsk3b-Mm00444911_m1	Kinase	No change
3-hydroxyacyl-CoA dehydrogenase type-2	Hadh2-Mm00840109_m1	Other	No change
Insulin degrading enzyme	Ide-Mm00473077_m1	Peptidase	No change
Interferon gamma	Ifng-Mm00801778_m1	Cytokine	No change
Interleukin 1 alpha	Il1a-Mm00439620_m1	Cytokine	**Down regulated**
Interleukin 1 beta	Il1b-Mm00434228_m1	Cytokine	No change
Interleukin 6	Il6-Mm00446190_m1	Cytokine	No change
Insulin I	Ins1-Mm01950294_s1	Other	No change
Insulin receptor	Insr-Mm00439693_m1	Receptor	No change
LDL receptor related protein 1	Lrp1-Mm00464608_m1	Receptor	No change
LDL receptor related protein 2	Lrp2-Mm01328171_m1	Receptor	No change
LDL receptor related protein associated protein 1	Lrpap1-Mm00660272_m1	Receptor	No change
Mitogen-activated protein kinase 1	Mapk1-Mm00442479_m1	Kinase enzyme	**Up regulated**
Mitogen-activated protein kinase 3	Mapk3-Mm00662375_g1	Kinase enzyme	No change
Microtubule associated protein tau	Mapt-Mm00521988_m1	Other	No change
Membrane metalloendopeptidase	Mme-Mm00485028_m1	Peptidase	No change
Nicastrin	Ncstn-Mm00452010_m1	Other	No change
Phosphodiesterase 8B	Pde8b-Mm01283824_m1	Enzyme	No change
Protein kinase N1	Pkn1-Mm00723995_m1	Kinase enzyme	No change
Phospholipase D1	Pld1-Mm01289339_m1	Enzyme	No change
Protein phosphatase 2 catalytic subunit alpha	Ppp2ca-Mm00479816_m1	Enzyme	No change
Protein kinase cAMP-activated catalytic subunit beta	Prkacb-Mm00440840_m1	Kinase enzyme	No change
Protein kinase C alpha	Prkca-Mm00440858_m1	Kinase enzyme	No change
Protein kinase cAMP-activated catalytic subunit beta1	Prkcb1-Mm00435749_m1	Kinase enzyme	No change
Protein kinase C, gamma gene	Prkcc-Mm00440861_m1	Kinase enzyme	No change
Protein kinase C epsilon	Prkce-Mm00440894_m1	Kinase enzyme	No change
Presenilin 1	Psen1-Mm00501184_m1	Transmembrane receptor	No change
Presenilin 2	Psen2-Mm00448405_m1	Transmembrane receptor	No change
Presenilin Enhancer, gamma secretase subunit	Psenen-Mm00727761_s1	Enzyme	No change
Serine (or cysteine) peptidase inhibitor, clade A, member 3N	Serpina3n-Mm00776439_m1	Enzyme	No change
Solute carrier family 30-member 3	Slc30a3-Mm00442148_m1	Solute carrier	No change
Synuclein alpha	Snca-Mm01188700_m1	Other	No change
Superoxide dismutase 2	Sod2-Mm00449726_m1	Enzyme	No change
ST6 beta-galactoside alpha-2,6-sialyltransferase 1	St6gal1-Mm00486119_m1	Enzyme	No change
Transcription factor AP-4	Tcfap4-Mm00473137_m1	Transcription factor	No change
Tumour necrosis factor	Tnf-Mm00443258_m1	Cytokine	No change
Ubiquilin 1	Ubqln1-Mm01334970_g1	Peptidase	No change
Ubiquitin C-terminal hydrolase L1	Uchl1-Mm00495900_m1	Enzyme	No change
visinin like 1	Vsnl1-Mm00449558_m1	Other	**Up regulated**
Actin, Beta, cytoplasmic	actb-Mm00607939_s1	Other	No change
Hypoxanthine guanine phosphoribosyl transferase 1	hprt1-Mm00446968_m1	Enzyme housekeeping gene	N/A
Importin 8	ipo8-Mm01255158_m1	Transport receptor and/or as an adapter-like protein housekeeping gene	N/A

Following qPCR, each of the tissue samples were analyzed per card for the 96 genes. The expression of all genes was determined using the 2- ΔΔCt method.^27^ Using this method, the fold-changes were obtained in *P. gingivalis* infected (test samples) gene expression, which were normalized to an internal control housekeeping gene (18S). The data was expressed relative to the sham-infected (control brain tissue samples) at each time (12- and 24-weeks) points.

### Statistical analysis

Fold change in the expression of AD genes was determined by qPCR analysis of the ApoE^−/−^ mice brains with confirmed *P. gingivalis* DNA within the brains (Table 1) across the sham-infected controls. A two variant T-test (T-Test) was carried out using the Excel program. A *p* value, less than or equal to ≤0.05 was considered significant.

## Results

### Taqman™ mouse Alzheimer disease arrays

To reiterate, *P. gingivalis* DNA isolated from the infected mouse brains was identified by PCR in the Poole et al.^12^ study.

Of the four housekeeping genes, 18S rRNA (18S) (Table 2) gave the most stable expression within all the samples analyzed. Hence all data (*P. gingivalis* infected and sham infected animals) was initially normalized to the 18S housekeeping gene and then test data was expressed relative to the sham-infected at 12- and 24-weeks post infection.

### 12-weeks post infection

Of the 92 AD associated genes (96 minus the four housekeeping genes) evaluated in the *P. gingivalis* 12-weeks post infection, the key target genes with statistically significant changes of their expression were cyclin dependent kinase 5 regulatory subunit 1 (Cdk5r1, 0.15 logfold change, p = 0.05) and Interleukin 1 alpha, (IL1a, −0.10 log fold change, p = 0.012). No change was observed in the other 90 out of 92 AD genes at this time point (Table 2). Data presented was expressed relative to the sham-infected at 12-weeks post infection (Figure 1).

### 24-weeks post infection

**Figure 2.**
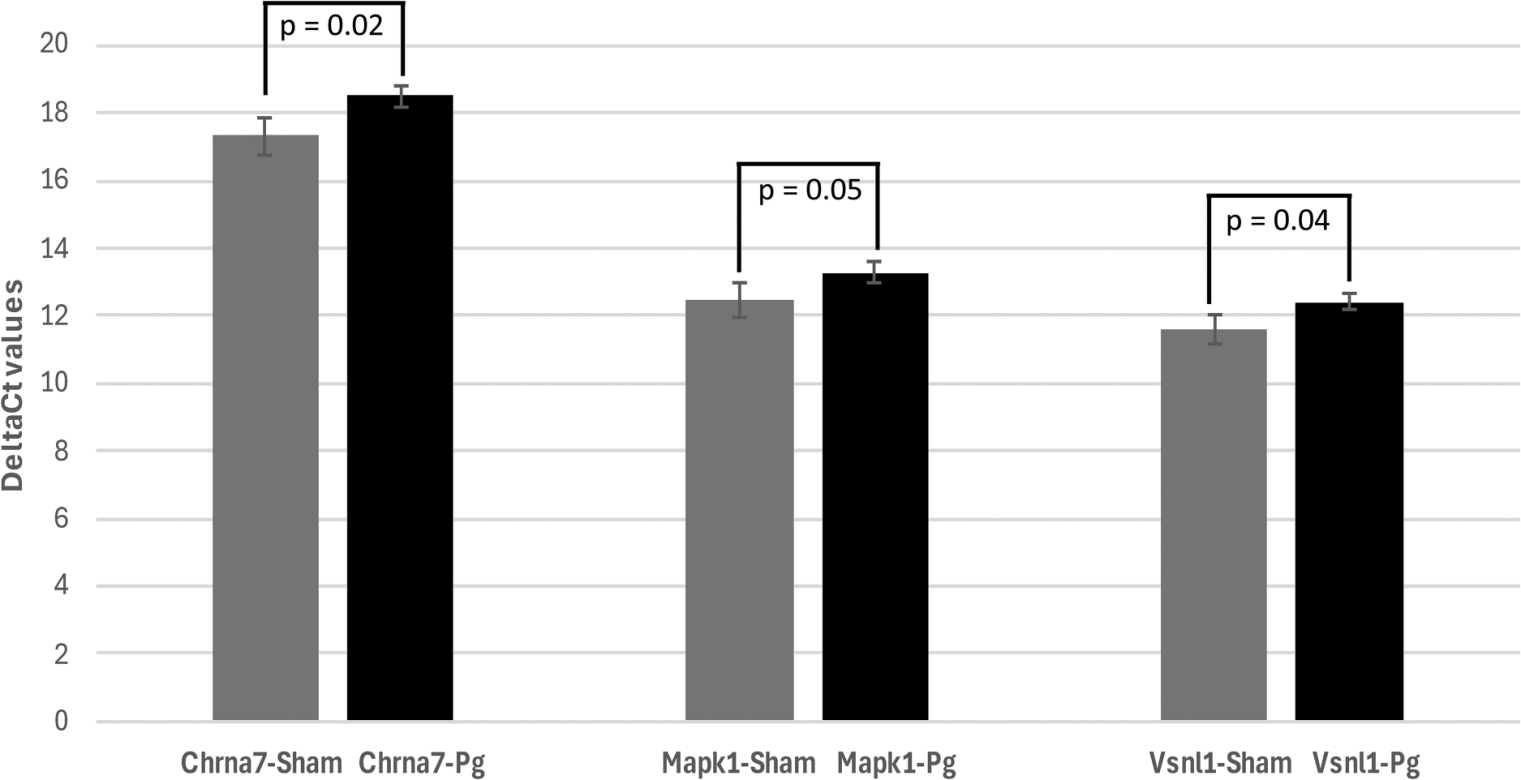
q-PCR analysis 24-weeks post infection. Of the 92 target genes evaluated in the *P. gingivalis* 24-weeks post infection the key genes that were statistically significantly upregulated by their expression were Chrna7 (0.10 log fold change, p = 0.02), Mapk1 (0.10 log fold change, p = 0.05) and Vnsl1 (0.01 log fold change, p = 0.04). The expression of the three genes by q-PCR analysis (N = 4) across the sham infected (control) and *P. gingivalis* (Pg) infected was analyzed by the two variant T-Test.

Of the 92 AD associated genes evaluated in the *P. gingivalis* 24-weeks post infection the key genes with statistically significant changes of their expression were cholinergic receptor nicotinic alpha 7 subunit or Chrna7 (0.10 log fold change, p = 0.02), mitogen-activated protein kinase 1 or Mapk1 (0.10 log fold change, p = 0.05) and visinin like 1 or Vnsl1 (0.01 log fold change, p = 0.04). No change was noted in the remaining 89 out of 92 AD genes (Table 2). Data presented was expressed relative to the sham-infected at 24-weeks post infection (Figure 2).

## Discussion

The National Institute on Aging proposes advancing age, genetics, health (diabetes, hypertension, periodontitis) and recurrent urinary tract and pneumonia all contribute to increasing the risk of developing AD. In this context, periodontitis is a modifiable condition, and addressing it aligns with future prevention plans for AD.^28^

This study concentrated on identifying AD genes stimulated in ApoE^−/−^ mice, by orally infected with *P. gingivalis* (FDC 381) with the presence of the bacterium in the brain confirmed by PCR in the initial investigation by Poole et al.^12^ That study was a ‘proof of concept showing *P. gingivalis* translocated from the mouth to the brain, and the brain tissue analyzed in the recent study was from confirmed *P. gingivalis* DNA as previously reported.^12^ However, any specific effect of the bacterium on the formation/number of Aβ plaques and NFTs proved to be inconclusive,^12^ although the deleterious effects of the bacterium could include oxidative damage^13^ of the cerebral and microvasculature with marginally increased activation of microglia and astrocytes, and not just NFT-like formation.

With the commercial availability of the TaqMan™ Mouse Alzheimer disease arrays, we used gene array methodology to identify which AD genes were stimulated by *P. gingivalis* following its entry into the brain tissue. Gene expression analysis of a panel of 96 genes of which (92 comprised of AD associated genes and four other housekeeping genes, in which the latter genes were essential for normalizing the data) was performed. At 12-weeks post infection, only two AD genes Cdk5r1 and IL1a demonstrated statistically significant alteration of their expression compared with the sham-infected brain tissue.

To contextualize these findings, Cdk5r1 is a gene essential for the development of the central nervous system (CNS). It was upregulated by *P. gingivalis* infection in the ApoE^−/−^ mice after 12 weeks.^29,30^ However, altered Cdk5 activity is relevant to neurodegeneration mediated by its neuron-specific activator protein, p35.^31^ This protein is cleaved into a smaller fragment p25,^32,33^ facilitating its entry into the neuronal nucleus,^34^ to prolong the activation of Cdk5.^35^ A longer term consequence of Cdk5 upregulation is increased aberrant phosphorylation of the tau protein, an important constituent of the paired helical filaments found in NFTs.^36^

Additionally, pathways associated with Cdk5 signal transduction also modulate the circadian rhythm,^37–40^ which is adversely affected in AD patients.^39^ To our knowledge, brain infection with *P. gingivalis*, has not been reported to be associated with CDK5 upregulation involving the activation of specific circadian rhythm pathway genes/proteins. In a wider context IL1a gene expression decreased, and this reflects a poorly regulated immune system suppressing inflammation. In cases of delirium associated with systemic infections, different cytokines (IL1a unlikely) are involved.^41^ In the context of periodontal disease, Takayama et al.^42^ noted *P. gingivalis* infection of mice caused sleep pattern disturbances, attributed to altered glial cell light/dark molecular clock activity. While it is clear that the hypothalamic-pituitary-adrenal axis plays a role in the maintenance of sleep/wake patterns, there is some evidence to link sleep cycle disturbance with systemic infections and delirium through the influence of CDK5 gene expression in the hypothalamus. This has, to some extent, been reported in that hypothalamus is enriched in genes attributed to *P. gingivalis* infection in mice brains.^43^

Surprisingly the IL1a gene was downregulated by *P. gingivalis* infection in the ApoE^−/−^ mice brain at 12 weeks increasing the vulnerability of the CNS to pathogen invasion. This outcome corroborates with the report by Poole et al.^12^ showing activated microglia and astrocytes were present around the lateral ventricles and within the hippocampus (i.e., the pathogen was likely present in the cerebrospinal fluid) without reaching statistical significance. The specific down regulation of the ILla cytokine gene in the ApoE^−/−^ mice at 12 weeks is consistent with the increased vulnerability of this AD mouse genotype to systemic infection.

At 24-week post infection, the AD genes Chrna7, Vnsl1, and MAPK were upregulated, and they play significant roles in the homeostatic function of neurons. Chrna7, an alpha 7-nicotinic acetylcholine receptor protein (α7-nAChRs) is involved in chemical and electrical signaling, neurite outgrowth, synaptic plasticity neuronal death and/or survival all relevant as an acute neuronal repair response following systemic infection.^44^ Synaptic dysfunction is one of the earliest structural defects associated with declining memory.^45–47^ The efficacy of the electrical signal transmission via synaptic junctions is activity-dependent and is continuously modified during development and adult life.^48^ Therefore, the expression of Chrna7 gene is highly relevant for maintaining normal neuronal function when exposed to systemic infections; future research should explore its significance in the context of ApoE gene mutation and *P. gingivalis* infection.

Vnsl1 modulates calcium binding pathways, linked to cellular processes directly associated with learning and memory within the hippocampus depending on the type of stimulus a neuron receives.^49^ Calcium-signaling proteins such as calcium-calmodulin kinase II, calcium-response element binding protein have a key role in both long term potentiation and long-term depression. The increased expression of Vnsl1 and Chrna7 could reflect early attempts at neuronal repair and plasticity in response to systemic infections including periodontitis. Wu et al.^50^ experimentally tested the effect of chronic *P. gingivalis* LPS exposure on memory for AD-like phenotypes in middle-aged (12 months old) mice, providing initial clues towards understanding cognitive impairment in a *P. gingivalis* LPS infected animal model. Wu et al.^50^ were the first to report that chronic exposure to LPS from *P. gingivalis* elicited AD-like phenotypes in middle-aged (12 months old) mice. The phenotypes included learning and memory deficits, intracellular Aβ in neurons, and microglia-induced neuroinflammation in the hippocampus and interleukin (IL)-1β with its cognate receptor (IL-1R) on neurons. Exposure to LPS from *P. gingivalis* led to a significant increase in microglia implying their activation and subsequent secretion of cytokines. A subsequent report by Zhang et al.^51^ conducted functional testing but specifically analyzing the effect of *P. gingivalis* LPS on cognitive function. Behavioral changes were assessed with the open field test, Morris water maze, and passive avoidance test. Using immunohistochemistry, they assessed for activation of astrocytes and microglia within the cerebral cortex and hippocampus; and assessed for pro-inflammatory cytokine expression of IL-1β, IL-6, IL-8, tumor necrosis factor-α (TNFα), toll like receptors (TLRs), and CD-14 using reverse transcriptase-polymerase chain reaction (RT-PCR), enzyme-linked immunoassay (ELISA), and western blotting. The *P. gingivalis*-LPS infected mice showed impairment in spatial learning and memory, along with activation of microglia and astrocytes within both the cerebral cortex and hippocampus. These findings are in accord with the impact of *P. gingivalis* infection mediated acute inflammation and the Vnsl1 and Chrna7 increased expression at early attempts of neuronal repair and plasticity in response to systemic infections. Noble et al.^52^ found that *P. gingivalis* infection was associated with impaired spatial/episodic memory in AD with an odds ratio of 2.00 (95% CI 1.19 to 3.36) after adjusting for confounders in the NHANES-III study supporting deteriorating memory following systemic infections.

Mapk1 regulates a range of gene expression related to multiple cellular processes mitosis, metabolism, cell motility, apoptosis and differentiation. The Mapk pathways comprise the extracellular signal-regulated kinase (ERK), c-Jun N-terminal kinase, and p38 proteins.^53,54^ Inadvertent activation of the Mapk pathways is implicated in AD^53,54^ and its proposed activator is Aβ.^55^ However, this was unlikely in these experiments as Aβ was not detected in these mice brains as confirmed by immunostaining.^12^ Notably a constituent of the *P. gingivalis* outer cell wall LPS, is a known activator of the p38, Mapk and ERK1/2 signaling pathways^56,57^ regulating inflammatory cytokines and stress related proteins as part of the host's inflammatory response (activated microglia and astrocytes) to pathogen invasion and or infection.^56,57^

The direct effect of the activated p38 kinase pathway is the deregulation of inflammation (e.g., reduced levels of TNF-α, IL-1, IL-6, and IL-8) maintaining prolonged brain inflammation. Prolonged oxidative stress episodes produce an excessive accumulation of reactive oxygen species, capable of inducing deleterious cellular and biomolecular damage to the host,^13,58–60^ and inadvertently activating GSK-3β,^61^ further increasing the possibility of tau phosphorylation. This finding strongly implicates the role of *P. gingivalis* LPS activity in activating GSK-3β,^61^ possibly via the Mapk pathways to promote reactive oxygen species and oxidative stress supporting inflammation and tau phosphorylation.^62^

### Limitations of the study

This study has focused on the effects of the periodontal pathogen *P. gingivalis* infection on gene expression in a mouse model of AD using the commercial TaqMan™ Mouse Alzheimer disease arrays. While gene expression indicates putative cellular changes, protein levels more accurately reflect changes in cellular functions and pathological processes. Therefore, both sets of experiments are necessary to study human AD pathology and the extrapolation of periodontal disease effects from rodents to humans.

### Conclusions

Identifying five AD associated genes CDK, IL-Ia, Chrna7, Vnsl1, and Mapk1) with statistically significant expression following *P. gingivalis* entry into the brain in the ApoE^−/−^ mice is crucial for the understanding the role of periodontal infection in the early pathogenesis of AD. This finding is significant as it shows periodontal disease (as an example of systemic infection) has selective effects upon the regulation of genes associated with inflammation and early changes related to neuronal plasticity and repair. These *in vivo* data are relevant to understanding the underlying cellular dysfunction in AD.

If adequate oral hygiene is not maintained periodontitis will become a two-five fold risk factor for the development of AD.^63^ Periodontitis is a modifiable condition, and addressing it makes sense to prevent or delay the onset of AD later in life. Therefore, future research should focus on determining the mechanisms of *P. gingivalis* infection in relation to the AD common genes identified here.
